# Special Issue “Molecular Mechanism Discovery of the Bioactive Phytochemicals Against Different Diseases Based on Network Pharmacology and Molecular Docking, 2nd Edition”

**DOI:** 10.3390/ijms27219619

**Published:** 2026-10-27

**Authors:** Baojun Xu

**Affiliations:** Food Science and Technology Program, Department of Life Sciences, Beijing Normal-Hong Kong Baptist University, Zhuhai 519087, China; baojunxu@bnbu.edu.cn; Tel.: +86-756-3620636

## 1. Preface

Plant-derived bioactive phytochemicals represent an invaluable resource as lead compounds for disease prevention and therapeutic treatment [1], attracting interest across the fields of food science, nutraceutical research, and drug discovery. Over the past decade, research paradigms for phytochemicals have transitioned from conventional isolation and biological activity validation toward the systematic examination of multitarget molecular mechanisms underlying their health-regulating effects [2]. Network pharmacology paired with molecular docking simulation has emerged as a powerful integrative toolkit to decode complex compound–target–disease interactions, enabling high-throughput hypothesis generation and prioritization of bioactive constituents for downstream experimental validation [3].

This Special Issue, “Molecular Mechanism Discovery of the Bioactive Phytochemicals Against Different Diseases Based on Network Pharmacology and Molecular Docking, 2nd Edition”, published in *International Journal of Molecular Sciences*, continues the success of first edition. It aimed to gather pioneering original research and comprehensive reviews applying network pharmacology, molecular docking, and complementary bioinformatic workflows to uncover the molecular mechanisms of phytochemicals to address diverse human diseases. Manuscript submissions covering natural products, medicinal herbs, edible mushrooms, functional food ingredients, and their multitarget therapeutic actions were encouraged. Following rigorous peer-review evaluation, six high-quality contributions, including five original research articles and one review paper, were published in this Special Issue, mirroring current advances and practical limitations of in silico-driven phytomedicine investigation.

## 2. Overview of the Contributions

The six published manuscripts span a broad disease spectrum including diabetes mellitus, androgenetic alopecia, melanoma, prostate cancer, and other malignant conditions. They integrate LC-MS phytochemical profiling, network pharmacology target prediction, protein–ligand molecular docking, and public-dataset bioinformatics mining, as well as in vitro and in vivo experimental validation to characterize bioactive natural compounds.

### 2.1. Phytochemicals for Metabolic Disorders

Shah et al. (2025) [Contribution 1] investigated the antidiabetic potential of *Cichorium intybus* (chicory), a well-known functional food and coffee substitute. Guided by an α-glucosidase inhibitory in vitro assay, the authors combined network pharmacology and molecular docking to pinpoint core bioactive metabolites and their type-2-diabetes-associated targets and signaling cascades. Their findings offer mechanistic support for chicory as a promising source of natural anti-hyperglycemic agents.

Randeni et al. (2025) [Contribution 2] examined the antidiabetic molecular mechanisms of the medicinal mushroom *Inonotus obliquus* (Chaga). A total of 30 bioactive constituents dominated by triterpenoids were identified using UHPLC-QE-MS. Network pharmacology highlighted 167 core antidiabetic targets enriched in the PI3K/Akt signaling axis. Molecular docking verified a favorable binding affinity between representative compounds and key disease-relevant proteins, while transcriptome-based public dataset analysis and immune-cell infiltration analysis further supplemented the mechanistic interpretation.

### 2.2. Natural Products for Dermatological Conditions

Subedi et al. (2025) [Contribution 3] evaluated the hair-regenerative efficacy of broccoli sprout extract (BSE) in the treatment of androgenetic alopecia (AGA). In vitro cellular assays demonstrated proliferative and migratory benefits on skin-derived cells. In a testosterone-induced AGA mouse model, oral BSE markedly accelerated hair regrowth. Mechanistic exploration indicated that BSE modulated hepatic and cutaneous dihydrotestosterone-catabolizing enzymes and triggered Wnt/β-catenin skin signaling. In silico docking corroborated the favorable binding of principal extract components to relevant target proteins.

### 2.3. Antitumor Phytochemical Investigations

Darilag et al. (2025) [Contribution 4] explored bioactive compounds derived from *Bambusa stenostachya* leaf material against melanoma. Seven phytochemicals satisfying Lipinski’s druglikeness criteria were characterized using LC-MS. Network pharmacology uncovered PIM1, MEK1, CDK2, and PDK1 as prospective antimelanoma therapeutic targets. Ensemble molecular docking demonstrated the strong ligand–protein interaction of naringin-7-rhamnoglucoside against these oncogenic targets, implying that the bamboo leaf metabolites may constrain melanoma progression by reprogramming oncogenic signaling pathways.

Austria et al. (2025) [Contribution 5] assessed the anti-prostate-cancer properties of aqueous and ethanolic extracts from *Rosmarinus officinalis* (rosemary) leaf. In vitro cytotoxicity against DU-145 prostate carcinoma cells revealed solvent-dependent bioactivity variance. Network pharmacology pinpointed key oncogenic genes, and molecular dynamics plus binding energy calculation demonstrated stable interactions of carnosol and rosmarinic acid with prostate-cancer-related protein targets, providing computational evidence for rosemary’s chemopreventive capacity.

Jang et al. (2025) [Contribution 6] contributed a comprehensive review focusing on multitarget anticancer mechanisms of acacetin, a natural flavonoid. They summarized the existing in vitro and in vivo tumor-model evidence of acacetin and systematically synthesized network-pharmacology and docking-derived predictions together with experimental validations, consolidating EGFR, STAT3 and AKT as central effectors mediating acacetin’s anticancer activities. They also discussed translational challenges and provided a future development outlook for acacetin-related antitumor research.

## 3. Outlook

Collectively, these six publications illustrate the strengths and existing bottlenecks of network pharmacology plus molecular docking workflows in phytochemical-oriented disease research. In silico strategies enable rapid hypothesis generation for multi-target mechanisms of complex natural-product mixtures; nevertheless, computational predictions urgently demand adequate wet-lab experimental verification to avoid the over-interpretation of pure simulation outcomes.

Key prospective research directions include the following: (1) improvement in target-prediction algorithms tailored for plant/fungal secondary metabolites; (2) standardized multi-omics experimental validation to cross-validate network pharmacology outputs; (3) construction of compound combination models to reflect synergistic actions of natural-product mixtures; (4) integration of artificial intelligence tools to optimize molecular docking performance for natural small molecules; (5) advancing from cell level assays towards animal model and preclinical studies to translate computational discoveries into nutraceutical or pharmaceutical applications.

## Data Availability

No new data were created or analyzed in this study. Data sharing is not applicable to this article.
